# 奥妥珠单抗短程输注中国专家共识（2024年版）

**DOI:** 10.3760/cma.j.cn121090-20240704-00250

**Published:** 2024-10

**Authors:** 中国抗癌协会血液肿瘤专业委员会 中华医学会血液学分会

## Abstract

奥妥珠单抗自上市10余年来，广泛应用于CD20阳性B细胞非霍奇金淋巴瘤患者并带来显著临床获益。目前国内外已有研究证实，奥妥珠单抗90 min短程输注方案的安全性好、可行性佳。为提高患者输液的便利性和提升医疗机构输液设施的使用效率，推荐对第1个周期奥妥珠单抗按标准速率输注未发生≥3级输液相关不良反应的患者，后续疗程采用短程输注的方案。国内相关领域专家经过充分讨论制定本共识，以期为临床实践提供指导。

## Abstract (en)

Obinutuzumab has been widely used in patients with CD20 positve B-cell non-Hodgkin's lymphoma for more than 10 years since its launch, bringing significant clinical benefits. At present, studies at home and abroad have confirmed that the 90-minute short duration infusion of obinutuzumab is safe and feasible.In order to improve the convenience of patient infusion and the efficiency of medical institutions' infusion facilities, it is recommended for patients who do not experience grade ≥3 infusion-related adverse reactions in the first cycle of obinutuzumab infusion at the standard rate, a short duration infusion scheme should be used for subsequent treatment courses. This expert consensus is formulated to provide guidance for clinical practice.

奥妥珠单抗注射液（obinutuzumab injection）是一种新型人源化抗CD20单抗，该药于2013年7月经美国食品药品管理局（FDA）批准上市，用于初治慢性淋巴细胞白血病（CLL）、初治或复发难治滤泡性淋巴瘤（FL）及后续维持治疗[1]。2021年6月奥妥珠单抗获得中国国家药品监督管理局（NMPA）批准，用于初治Ⅱ期伴有巨大肿块、Ⅲ期或Ⅳ期FL，以及达到至少部分缓解FL患者的后续维持治疗[2]。此外，奥妥珠单抗在弥漫大B细胞淋巴瘤、套细胞淋巴瘤、边缘区淋巴瘤等多种B细胞非霍奇金淋巴瘤（NHL）中亦有广泛应用和推荐[3]–[4]。输液相关不良反应（infusion-related reaction，IRR）是奥妥珠单抗最常见的药物治疗相关不良事件，主要发生在首个1 000 mg剂量输注期间。为了减少IRR的发生，按照药品说明书的推荐缓慢递增输注速率，首次输注时长至少4.25 h（CLL约8 h）[5]。较长时间的输液会导致患者身心疲劳以及输液设施使用效率的降低。奥妥珠单抗相比利妥昔单抗具有更强的抗体依赖细胞毒性作用和吞噬作用，其能否进行短程输注（short duration infusion，SDI）备受关注。参照利妥昔单抗SDI的实践标准[6]，国外GAZELLE[7]–[8]、GATHER[9]–[10]、GATS[11]和Fleck C[12]等研究和国内部分医学中心[13]–[14]等研究发现，针对第1个周期奥妥珠单抗输注未发生≥3级IRR的患者，后续疗程进行奥妥珠单抗90 min SDI具有良好的安全性和可行性，且奥妥珠单抗的稳态药代动力学在第2个周期达到，不受SDI影响。同时，2022年FDA批准了奥妥珠单抗90 min SDI可用于第2个疗程及后续疗程。因此，基于现有循证医学证据，本专家共识对奥妥珠单抗90 min SDI方案进行系统总结，以期对国内临床实践提供指导。

一、用药剂量和药液配置方法

奥妥珠单抗推荐剂量为固定剂量1 000 mg静脉给药。根据不同的联合治疗方案确定1个周期为21 d或28 d，经过最初6个或8个周期含奥妥珠单抗的联合治疗，达到完全或部分缓解的患者可继续接受奥妥珠单抗单药维持治疗，每2个月1次，用药2年或至疾病进展[15]。其中，在第1个周期采用剂量密集型给药方式（即于第1、8、15天分别给予1 000 mg）可更快达到高靶点饱和，为目前临床使用奥妥珠单抗的最优剂量。

根据奥妥珠单抗说明书中推荐[2]：奥妥珠单抗应由医护人员使用无菌针头和注射器按照无菌操作规范进行制备，在含有无菌、无致热原的0.9％氯化钠溶液的聚氯乙烯（PVC）或非PVC聚烯烃输液袋（可使用250 ml的输液袋）中进行稀释，稀释后最终浓度应该在0.4～4.0 mg/ml，如抽取40 ml（含1 000 mg）奥妥珠单抗浓缩液加入0.9％氯化钠溶液210 ml中配置成4.0 mg/ml的药液。《奥妥珠单抗临床用药指导原则中国专家共识（2021年版）》[15]推荐，在CLL治疗中，常在第1个周期的第1天给药100 mg，根据输注情况在第2天（或第1天继续）给药900 mg以提高输注安全性。江苏省人民医院血液科淋巴瘤诊疗团队针对B细胞NHL患者在首个1 000 mg奥妥珠单抗用药时尝试了2种不同的药液稀释方式[14]：在队列1中，抽取4 ml（含100 mg）奥妥珠单抗浓缩液加入0.9％氯化钠溶液246 ml中配置成0.4 mg/ml的药液，抽取36 ml（含900 mg）奥妥珠单抗浓缩液加入0.9％氯化钠溶液214 ml中配置成3.6 mg/ml药液；在队列2中，抽取4 ml（含100 mg）奥妥珠单抗浓缩液加入0.9％氯化钠溶液96 ml中配置成1.0 mg/ml药液，抽取36 ml（含900 mg）奥妥珠单抗浓缩液加入0.9％氯化钠溶液464 ml配置成1.8 mg/ml药液。两种药液稀释方式均在现有说明书和指南中的推荐范围，也避免了患者在首次输注过程中因发生严重IRR而造成的药液浪费。本专家共识推荐，针对首个1 000 mg奥妥珠单抗用药时建议分成2个输液袋配置，之后的治疗周期可采用1个输液袋配置。

二、预防用药

为降低IRR风险，在进行奥妥珠单抗标准输注或SDI前均应给予预防用药，如预先使用糖皮质激素、解热镇痛药物、和（或）抗组胺药物（如异丙嗪、苯海拉明）等，具体见奥妥珠单抗说明书中推荐[2]。值得注意的是，说明书中预处理药物剂量目前尚未达成共识，仅作为示例给出，可根据患者实际情况或当地用药指南进行调整。江苏省人民医院血液科淋巴瘤诊疗团队比较了不同预处理方案对患者IRR的影响[14]：队列1患者每次用药前常规给予地塞米松5 mg静脉注射、异丙嗪25 mg肌肉注射以及对乙酰氨基酚250 mg口服。队列2患者在第1次用药前给予地塞米松5 mg静脉注射和异丙嗪25 mg肌肉注射，若患者未发生≥3级IRR，则后续每次用药前仅静脉注射5 mg地塞米松。结果显示，在首次用药时，接受口服对乙酰氨基酚预防的患者IRR发生率低于未接受口服对乙酰氨基酚预防的患者（10.72％对30.21％，*P*<0.001），但是在随后的输注中，两组患者的发生率差异无统计学意义（0.24％对0.26％，*P*>0.05）。这可能因为首次给药时患者肿瘤负荷较高导致更多的细胞因子释放，随着用药次数增加，体内靶细胞逐渐减少从而耐受药物反应[16]–[17]。本专家共识推荐，建议遵循奥妥珠单抗说明书中的推荐进行预防用药，针对首个1 000 mg剂量输注期间未发生≥3级IRR的患者，后续用药前是否减少预处理用药有待进一步验证。

三、标准输注方案

奥妥珠单抗第1个周期的首个1 000 mg剂量输注期间，应按照标准输注速率给药，全程使用心电监护仪严密监测患者心率、血压、血氧饱和度。关于FL和CLL患者在输注奥妥珠单抗不存在IRR或超敏反应情况下的标准输注速率以及在先前输注发生IRR情况下的具体建议见表1和表2。

**表1:** 滤泡性淋巴瘤的奥妥珠单抗标准输注方案

周期（个）	治疗日	总剂量（mg）	输注速率^a^
1	第1天	1 000	以50 mg/h的速率给药。输液速率可每30分钟提高1次，每次50 mg/h，最大速率为400 mg/h
1	第8、15天	1 000	若先前以最终输注速率≥100 mg/h输注期间，未出现IRR或出现1级IRR，则可以100 mg/h的速率开始输注，每30分钟提高100 mg/h，最大速率为400 mg/h； 若患者先前输注期间出现2级或更高级别的IRR，则以50 mg/h的速率给药，输液速率可每30分钟提高1次，每次50 mg/h，最大速率为400 mg/h
2～6或2～8；维持治疗（每2个月为1个周期，最长达2年或直至出现疾病进展）	第1天	1 000	若先前以最终输注速率≥100 mg/h输注期间，未出现IRR或出现1级IRR，则可以100 mg/h的速率开始输注，每30分钟提高100 mg/h，最大速率为400 mg/h； 若患者先前输注期间出现2级或更高级别的IRR，则以50 mg/h的速率给药，输液速率可每30分钟提高1次，每次50 mg/h，最大速率为400 mg/h

**注** IRR：输液相关不良反应；^a^只要患者能够耐受，输注速率可以递增

**表2:** 慢性淋巴细胞白血病的奥妥珠单抗标准输注方案

周期（个）	治疗日	总剂量（mg）	输注速率^a^
1	第1天	100	4小时内以25 mg/h的速率给药，勿增加输液速率
1	第2天（或第1天继续）	900	若先前输注期间未出现IRR，则以50 mg/h的速率给药。输液速率可每30分钟提高1次，每次50 mg/h，最大速率为400 mg/h； 若患者先前输注期间出现IRR，则以25 mg/h的速率开始给药，输注速率可每30分钟提高1次，每次最多提高50 mg/h，最大速率为400 mg/h
1	第8、15天	1 000	若先前以最终输注速率≥100 mg/h输注时，未出现IRR，则可以100 mg/h的速率开始输注，每30分钟提高100 mg/h，最大速率为400 mg/h； 若患者先前输注期间出现IRR，则以50 mg/h的速率给药，输液速率可每30分钟提高1次，每次50 mg/h，最大速率为400 mg/h
2~6	2~6	1 000	同上

**注** IRR：输液相关不良反应；^a^只要患者能够耐受，输注速率可以递增

四、SDI方案

在第1个周期静脉输注奥妥珠单抗未发生≥3级IRR的患者，在后续治疗周期可开展奥妥珠单抗90 min SDI方案[7]–[14]，以FL为例，具体见表3。

**表3:** 滤泡性淋巴瘤的奥妥珠单抗90 min短程输注（SDI）方案

周期（个）	治疗日	总剂量（mg）	输注速率^a^
1	第1天	1 000	以50 mg/h的速率给药。输液速率可每30分钟提高1次，每次50 mg/h，最大速率为400 mg/h
1	第8天^b^、第15天	1 000	若先前以最终输注速率≥100 mg/h输注期间，未出现IRR或出现1级IRR，则可以100 mg/h的速率开始输注，每30分钟提高100 mg/h，最大速率为400 mg/h； 若患者先前输注期间出现2级或更高级别的IRR，则以50 mg/h的速率给药，输液速率可每30分钟提高1次，每次50 mg/h，最大速率为400 mg/h
2～6或2～8；维持治疗（每2个月为1个周期，最长达2年或直至出现疾病进展）	第1天	1 000	如果在第1周期内未发生≥3级IRR，前30 min以100 mg/h的速率给药，之后60 min以900 mg/h速率给药； 如果在SDI期间发生1～2级IRR伴持续症状或发生3级IRR，则以标准输注速率给药

**注** IRR：输液相关不良反应；^a^只要患者能够耐受，输注速率可以递增；^b^若有充足用药经验和管理经验，可以在第2次1 000 mg用药时开始90 min SDI[13]–[14]

五、SDI适合人群

CD20阳性的B细胞NHL患者在第1个周期使用奥妥珠单抗标准输注方案中未发生≥3级IRR时，在后续治疗中可接受奥妥珠单抗90 min SDI方案。以下患者不适合进行奥妥珠单抗90 min SDI：①首次静脉输注奥妥珠单抗的B细胞NHL患者；②首次使用奥妥珠单抗标准输注方案过程中出现≥3级IRR患者；③高肿瘤负荷患者：巨大肿块（长径>7 cm）和（或）循环淋巴细胞计数>5×10^9^/L的患者；④心、肺、肾等脏器严重功能不全的患者；⑤年老体弱或其他不能耐受SDI的患者；⑥高敏感体质患者。

六、IRR的临床表现及处理

由于奥妥珠单抗经过糖基化工程结构改造，与效应细胞上的FcγⅢ受体亲和力增强，导致细胞因子释放可能性增加，这可能是奥妥珠单抗输注尤其是首次输注时IRR发生率高的原因[16]–[18]。大多数与单抗相关的输液不良反应通常在输注开始的30～120 min出现，从轻微的脸红等流感样症状到潜在的致命事件都可能发生[18]。IRR相关症状包括恶心、呕吐、腹泻、发热、寒战等，其他症状还包括呼吸系统症状（如支气管痉挛、咽喉刺激、哮鸣、喉水肿）和心脏症状（如心房颤动）[15]。依据2017年11月27日美国国家癌症研究所（NCI）发布的常见不良事件评价标准（CTCAE）5.0版对IRR的严重程度进行分级：1级：反应短暂且轻微，无需中断输液，无需治疗；2级：需要中断治疗或输液且迅速予以对症治疗，如应用抗组胺药、非甾体抗炎药等，需要≤24 h的预防用药；3级：状况处理延迟，如未立即对症状予以对症治疗和（或）短暂中断输液，初始症状改善后又复发，需住院治疗的临床后遗症；4级：危及生命，需要紧急治疗；5级：死亡。发生IRR时的输注速率调整建议见表4。应在有完备复苏设施的环境中进行奥妥珠单抗输注，应特别对既往有心脏或肺部疾病的患者和肿瘤负荷高的患者进行密切观测，一旦发现患者发生IRR，需积极给予对症处理。发生轻度IRR时，一般给予静脉注射地塞米松5 mg和（或）肌肉注射异丙嗪25 mg后，多数患者症状可缓解。若患者出现胸闷、气促或血氧饱和度≤95％，应立即给予双鼻导管或面罩吸氧。若患者出现恶心、呕吐，可指导其深呼吸、做吞咽动作，减轻恶心，并及时准备呕吐袋放置于患者周围。患者发生呕吐时，应指导患者尽量选择坐位，若只能平卧位，嘱患者头偏向一侧，防止其误吸，导致呛咳，甚至窒息。呕吐后，予温水漱口保持口腔清洁。呕吐严重者出现血压下降，则遵医嘱给予静脉输注复方氯化钠注射液500 ml扩容补液等对症处理。

**表4:** 针对不同分级输液相关不良反应（IRR）的奥妥珠单抗输注速率调整建议

IRR级别	处理
1～2级（轻度和中度）	降低输注速率或暂时中断输注，并对症治疗；待症状消退之后继续进行输注； 如果患者未再出现IRR，可按照治疗剂量增幅和时间间隔重新开始递增输注速率（表1、2）
3级（重度）	暂时中断输注并对症治疗； 在标准输注期间出现3级IRR的患者，在症状消退后，以不超过之前（IRR发生时的速率）一半的速率重新开始输注。如果没有出现进一步的IRR症状，可以按照对应周期治疗剂量的增幅和时间间隔重新开始递增输注速率（表1、2）； 对于在SDI期间发生3级IRR的患者，在症状消退后，以不超过之前速率（IRR发生时的速率）的一半且不超过400 mg/h的速率重新开始输注。如果患者能够完成输注且不再发生3级IRR，则以标准速率进行下一次输注； 如果患者再次发生3级IRR，则停止输注并永久性终止治疗
4级（危及生命）	停止输注并永久性终止治疗

**注** SDI：短程输注

七、总结

与标准速率输注相比，Fleck等[12]研究显示每次90 min SDI可节省输液椅时间94 min（1.56 h），在剩余疗程中每位患者节省时间中位数约为6.3 h，75次奥妥珠单抗90 min SDI共节省了117.5 h的输液椅时间。此外，90.6％的护士和86.3％的医师报告奥妥珠单抗90 min SDI可节省≥1 h的工作时间，15.7％的医师报告可节省≥4 h，且65.6％的护士和82.4％的医师认为奥妥珠单抗90 min SDI给药更方便，95.2％的护士和98.0％的医师首选奥妥珠单抗90 min SDI方案[8]。综上，针对第1个周期奥妥珠单抗按标准速率输注未发生≥3级IRR的患者，采用90 min SDI方案安全、可行且便利。随着奥妥珠单抗在我国的用药获批，患者治疗需求量激增，有必要在严密观察的前提下加强奥妥珠单抗SDI方案的进一步推广。
